# Caveolin-1 Y14 phosphorylation suppresses tumor growth while promoting invasion

**DOI:** 10.18632/oncotarget.27313

**Published:** 2019-11-19

**Authors:** Bharat Joshi, Judy Pawling, Jay Shankar, Karina Pacholczyk, Yohan Kim, Wynn Tran, Fanrui Meng, Anas M. Abdel Rahman, Leonard J. Foster, Hon S. Leong, James W. Dennis, Ivan R. Nabi

**Affiliations:** ^1^Department of Cellular and Physiological Sciences, Life Sciences Institute, University of British Columbia, Vancouver, Canada; ^2^Lunenfeld-Tanenbaum Research Institute, Mount Sinai Hospital, Department of Molecular Genetics, University of Toronto, Toronto, Canada; ^3^Translational Prostate Cancer Research Group, London Regional Cancer Program, University of Western Ontario, London, Canada; ^4^Department of Genetics, King Faisal Specialist Hospital and Research Centre (KFSHRC), Riyadh, Saudi Arabia; ^5^Centre for High-throughput Biology, University of British Columbia, Vancouver, Canada

**Keywords:** caveolin-1, TP53, tumor suppressor, tumor cell metabolism, invadopodia

## Abstract

Caveolin-1 is a transmembrane protein with both tumor promoter and suppressor functions that remain poorly understood. Cav1 phosphorylation by Src kinase on tyrosine 14 is closely associated with focal adhesion dynamics and tumor cell migration, however the role of pCav1 *in vivo* in tumor progression remains poorly characterized. Herein, we expressed phosphomimetic Y14D, wild type, and non-phosphorylatable Y14F forms of Cav1 in MDA-MB-435 cancer cells. Expression of Cav1Y14D reduced cell proliferation and induced the TP53 tumor suppressor. Ectopic expression in MDA-MB-435 cells of Y14 phosphorylatable Cav1 was required for induction of TP53 in response to oxidative stress. Cav1Y14D promotes an apparent reversal of the Warburg effect and markedly inhibited tumor growth *in vivo*. However, Cav1 induced pseudopodial recruitment of glycolytic enzymes, and time-lapse intravital imaging showed increased invadopodia protrusion and extravasation into blood vessels for Cav1WT and Y14D but not for Y14F. Our results suggest that Cav1 Y14 phosphorylation levels play a role in the conflicting demands on metabolic resources associated with cancer cell proliferation versus motility.

## INTRODUCTION

Caveolin-1 (Cav1) plays a complex role in cancer progression and has been ascribed both tumor promoter and suppressor functions; elevated Cav1 is associated with a poor prognosis in prostate, melanoma cancers and triple negative breast cancer [1, 2]. However, Cav1 induces premature senescence when over-expressed and is upregulated in aged fibroblasts [3]. Cav1 has been previously shown to induce TP53 expression [4, 5]. MDM2 is an E3 ubiquitin ligase that targets TP53 for proteasomal degradation and sequestration of MDM2 at the plasma membrane by Cav1 preserves nuclear TP53 activity [5]. Cav1 deficiency has also been reported to induce premature senescence via mitochondrial dysfunction [6], induce a switch from oxidative phosphorylation to glycolysis in response to oxidative stress [7] and mediate stromal cell regulation of mitochondrial metabolism of cancer cells [8]. Further, Cav1 increases aerobic glycolysis in cancer cells and promotes liver regeneration through hepatic glycolysis [9–11]. Cav1 therefore plays complex roles in the regulation of cancer cell metabolism [12].

In transformed cells as well as under oxidative stress conditions, Src kinase activity increases the phosphorylation of Cav1 on tyrosine 14 [13–15]. pCav1 is enriched in pseudopodial protrusions of tumor cells where it promotes focal adhesion turnover, focal adhesion signaling and cell motility [15–18]. ECM-induced Cav1 phosphorylation promotes melanoma invasion and metastasis [19, 20]. In this report, we characterize the effect of the dominant-active Cav1Y14D mutation on tumor growth and invasion *in vivo*. The MDA-MB-435 cancer cell line expresses very low levels of Cav1 and few caveolae [21], and herein serves as host for stable expression of wild-type, phosphomimetic Y14D and non-phosphorylatable Y14F Cav1 mutants [15, 17, 22]. Cav1Y14D induced TP53, reversed the Warburg effect and suppressed tumor growth, while supporting tumor cell invasion. These dual roles for pCav1 may in part account for previously reported tumor suppressor and progression activities of Cav1.

## RESULTS

### pCav1 induces a TP53 reversal of cancer cell proliferation, metabolism and tumor growth

**Figure 1:**
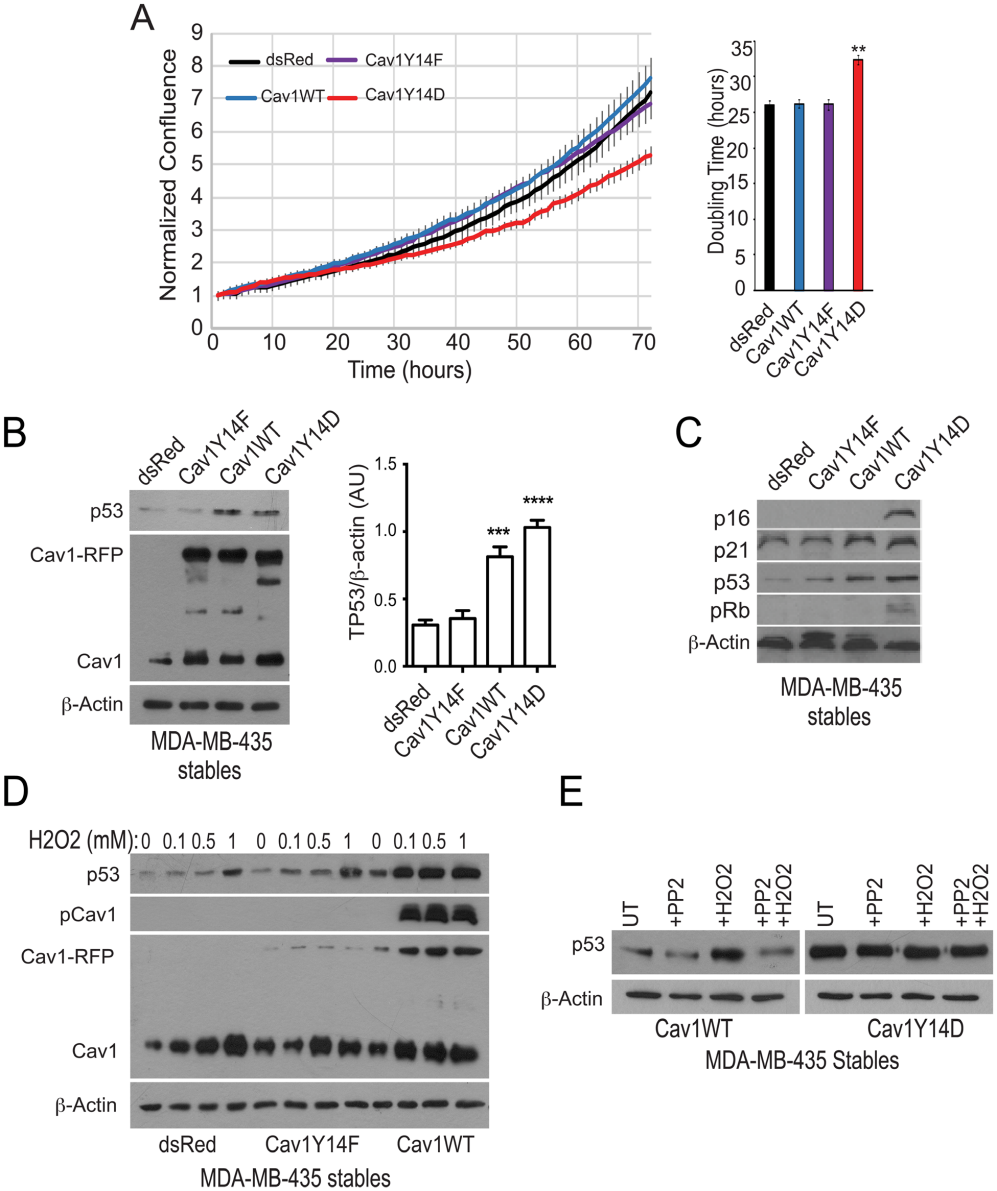
pCav1 restricts tumor cell proliferation and promotes TP53 expression. (**A**) Growth of MDA-MB-435 cells stably transfected with dsRed or RFP-tagged Cav1Y14F, Cav1WT or Cav1Y14D were imaged in an Incucyte chamber every hour over 70 hours and doubling time of the cells determined (^**^ *p* < 0.01; ANOVA). (**B**) MDA-MB-435 cells stably transfected with dsRed or RFP-tagged Cav1Y14F, Cav1WT or Cav1Y14D were Western blotted for TP53, Cav1 (showing both transfected Cav1-RFP and endogenous Cav1) and β-actin. Densitometric quantification of TP53 is shown relative to a β-actin loading control (*n* = 4, ^***^ *p* < 0.001, ^****^ *p* < 0.0001, ANOVA). (**C**) MDA-MB-435 cells stably transfected with dsRed or RFP-tagged Cav1Y14F, Cav1WT or Cav1Y14D were Western blotted for p16, p21, p53, pRb and β-actin. (**D**) MDA-MB-435 cells stably transfected with dsRed or RFP-tagged Cav1Y14F and Cav1WT were treated with indicated concentrations of H_2_O_2_. After treatment, cell lysates were Western blotted for TP53, Cav1 (showing both transfected Cav1-RFP and endogenous Cav1), pY14Cav1 and β-actin. (**E**) Stably transfected MDA-MB-435 cells, as indicated, were treated with the indicated amount of H_2_O_2_ (left) or with 0.1 mM H_2_O_2_ and/or 10 µM PP2 and cell lysates Western blotted for TP53 and β-actin.

MDA-MB-435 cell lines, that express the G266E gain-of-function TP53 mutation [23], were transfected with plasmids expressing either dsRed (control), wild-type Cav1, non-phosphorylatable Cav1Y14F or phosphomimetic Cav1Y14D [22]. Stable expression of Cav1WT and Cav1Y14F did not alter doubling time, however Cav1Y14D showed a significantly increased doubling time in cell culture (Figure 1A). TP53 levels were increased in Cav1WT and Cav1Y14D relative to dsRed and Cav1Y14F transfected MDA-MB-435 cells (Figure 1B) and TP53 effectors p16, p21 and pRb were elevated in Cav1Y14D cells (Figure 1C); Cav1 protein levels in Cav1Y14D cells were elevated (Figure 1B), as we previously reported [22]. Cav1 levels in these cell lines were previously shown to be similar to endogenous Cav1 levels in MDA-MB-231 breast carcinoma cells [22].

Oxidative stress stimulates Src-dependent Y14 phosphorylation of Cav1 [13, 14, 22]. In control MDA-MB-435 and Cav1Y14F cells, H_2_O_2_ treatment induced a minimal increase in TP53 compared to cells expressing Cav1WT (Figure 1D). Furthermore, Src inhibition with PP2 prevented induction of TP53 by H_2_O_2_ in Cav1WT MDA-MB-435 cells, and did not affect TP53 levels in phosphomimetic Cav1Y14D MDA-MB-435 cells (Figure 1E). Thus, ectopic expression of Cav1 and Y14 phosphorylation is required for oxidative stress-induced TP53 stabilization in MDA-MB-435 cells.

**Figure 2:**
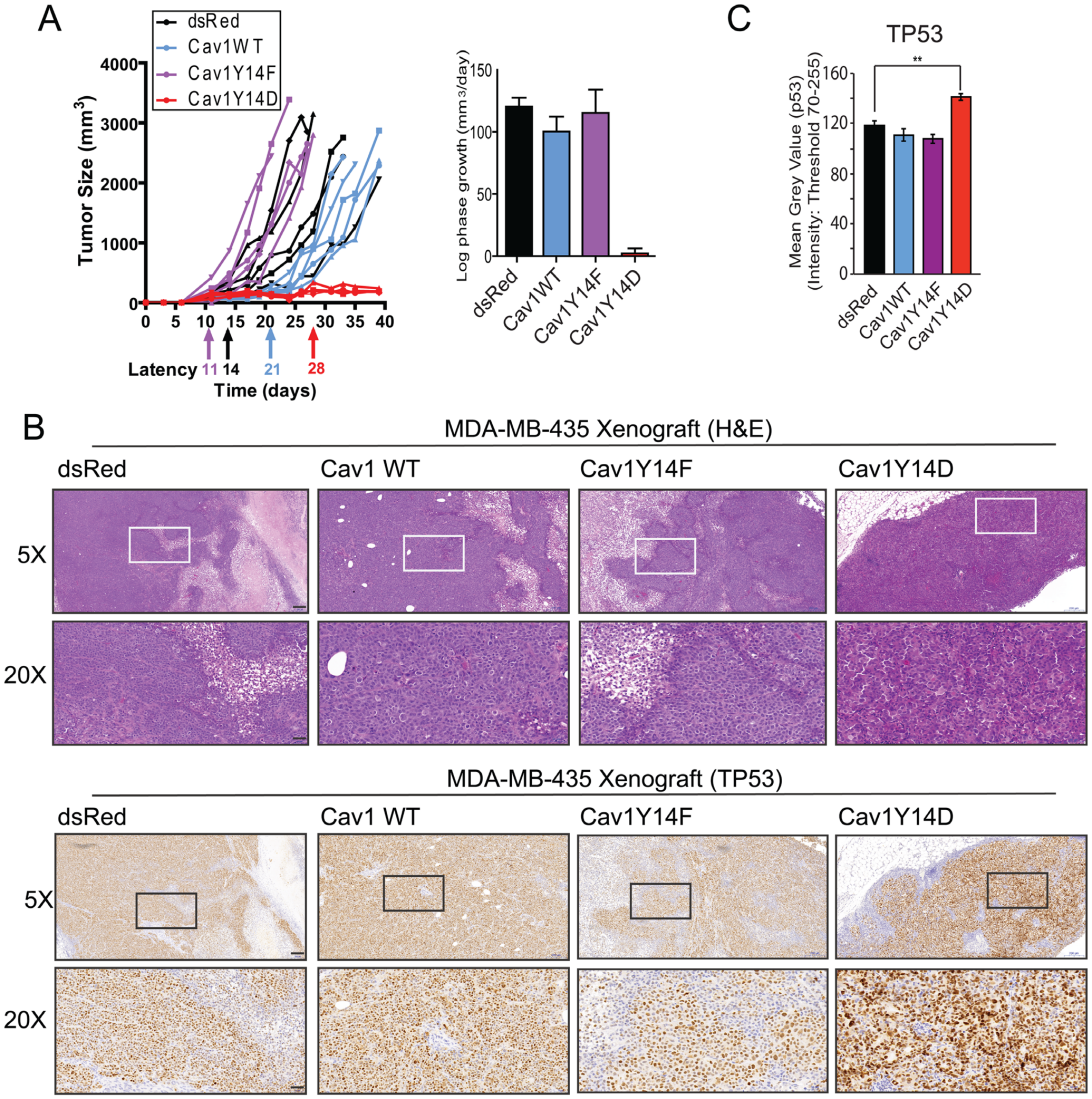
Tumor growth restriction of Cav1Y14D MDA-MB-435 cells. (**A**) Tumor growth of the MDA-MB-435 Cav1 Y14 stables following s.c. injection in nude mice was monitored; tumor size, latency and log phase growth are presented. (**B**) MDA-MB-435 tumors were labeled with H&E and immunostained for TP53. (**C**) Mean intensity of TP53 labeling in tumors was quantified (^**^ *p* < 0.01; ANOVA, Scale bar: 50 μm).

Subcutaneous injection into mice resulted in comparable log-phase tumor growth rates for dsRed, Cav1WT and Cav1Y14F tumor cells, whereas Cav1Y14D cells produced small palpable tumors, which failed to grow progressively (Figure 2A). Relative to dsRed tumor cells, tumor latency was shorter for Cav1Y14F and longer for Cav1WT cells. Immunohistochemistry staining revealed elevated nuclear TP53 in the small Cav1Y14D tumors at 4 weeks post injection (Figure 2B, 2C). This suggests that high-levels of Cav1Y14 phosphorylation slow cell proliferation and block tumor cell growth in a TP53-dependent manner in MDA-MB-435 cells.

**Figure 3:**
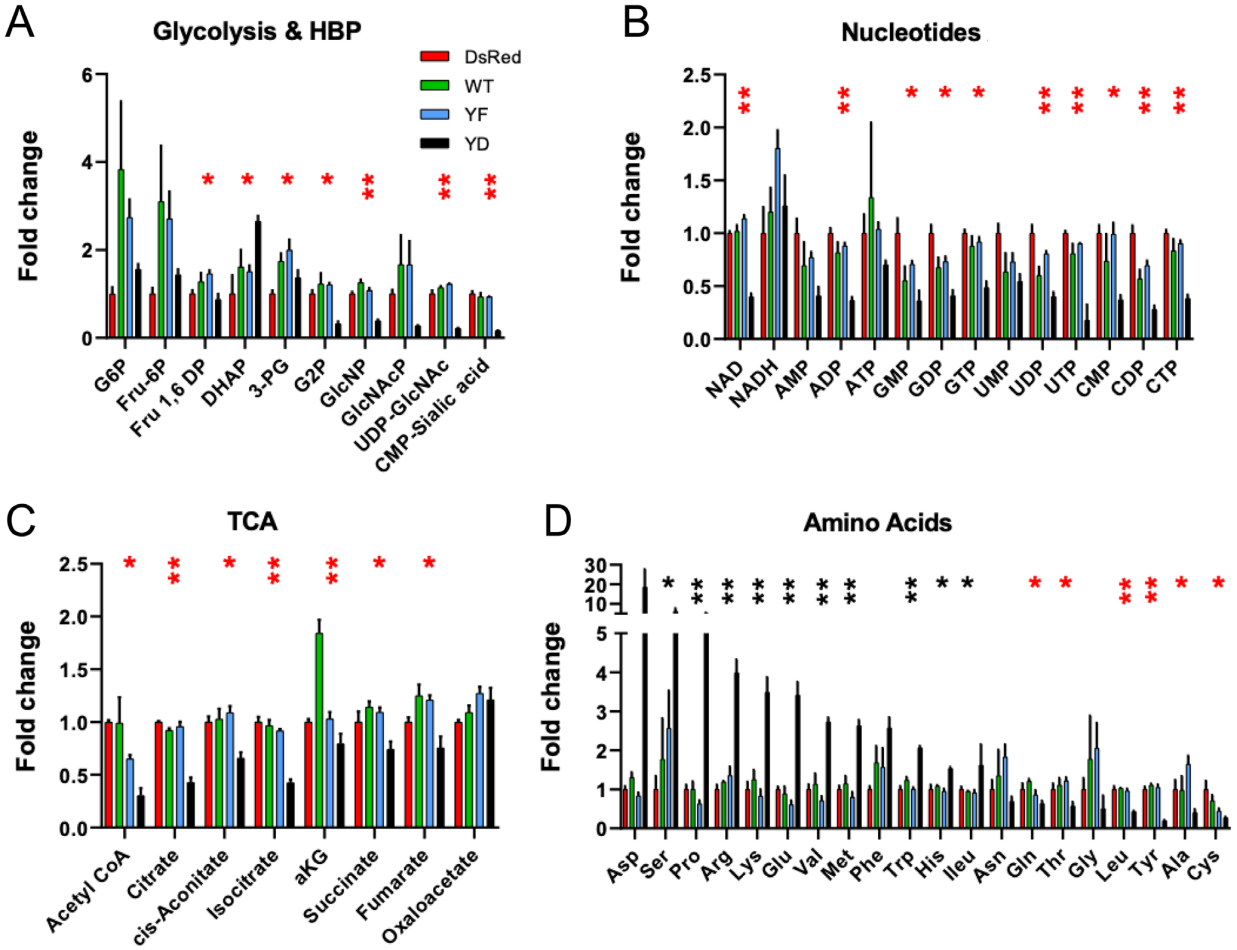
Metabolite profiles are consistent with slow-growth phenotype in Cav1Y14D tumors. Soluble metabolites were prepared from dsRed, Cav1Y14F, Cav1WT and Cav1Y14D MDA-MB-435 cells and quantified by LC-MS/MS as described in Methods. Fold change of glycolytic and hexosamine biosynthesis pathway (**A**), TCA cycle (**B**), nucleotide phosphates (**C**) and amino acids (**D**) was measured by LC-MS/MS. Glucose-6-phosphate (G6P); Fructose-1,6-phosphate (Fru1,6P); Fructose6-phosphate (Fru-6P); dihydroxyacetone phosphate (DHAP), 3-phosphoglyceric acid (3PG), 2-phosphoglyceric acid (G2P), N-acetylglucosamine phosphate (GlcNAcP); Uridine diphosphate N-acetylglucosamine (UDP-GlcNAc), and α-ketoglutarate (aKG). Error bars represent SD of three biological replicates (^*^ *p* < 0.05, ^**^ *p* < 0.001, ANOVA; black: Y14D > dsRed, red: Y14D < dsRed).

Loss of TP53 function in cancer cells leads to changes in gene expression and metabolism that support growth, whereas gain of TP53, as observed in Cav1Y14D cells, may reverse this metabolic phenotype [24, 25]. Analysis of intracellular metabolites distinguished Cav1Y14D cells from dsRed, Cav1WT and Cav1Y14F cells, consistent with high TP53 activity and slower growth. Decreased glucose-6-phosphate (G6P) is consistent with TP53-dependent suppression of glucose transporter expression (GLUTs) [26], thereby lowering fructose-6P (F6P) flux to glycolysis [24], the hexosamine biosynthesis pathway (HBP) and UDP-GlcNAc and CMP-sialic acid. In Cav1Y14D tumor cells, TCA cycle intermediates from acetyl-CoA to isocitrate are reduced, compared to those from α-ketoglutarate (α-KG) to oxaloacetate, suggesting that Gln catabolism is supporting oxidative phosphorylation [27], while flux from glucose to Ac-CoA is relatively lower (Figure 3A, 3B). Indeed, F6P as well as 3-phosphoglycerate (3PG), Ala and Gly were reduced in CavY14D cells, consistent with slowing of glycolysis and folate pathways, which are required for nucleotide biosynthesis and anti-oxidant response (Figure 3C). Cav1Y14D tumor cells displayed increases in Asp, Glu, Pro, Ser, Met, Lys and Arg, suggesting a delay or backup in their consumption by protein synthesis, purine biosynthesis, and Met in one-carbon transfer, Arg in nitrogen metabolism, and Lys and Pro linked to TCA cycle (Figure 3D). Together, the Cav1Y14D metabolite profile is consistent with a TP53-dependent suppression of growth. Nucleotide levels were lower in Cav1Y14D cells while energy charge (ATP/AMP+ADP) were unchanged, consistent with the specialized needs of motile cells.

### pCav1 induces invadopodia formation and extravasation

**Figure 4:**
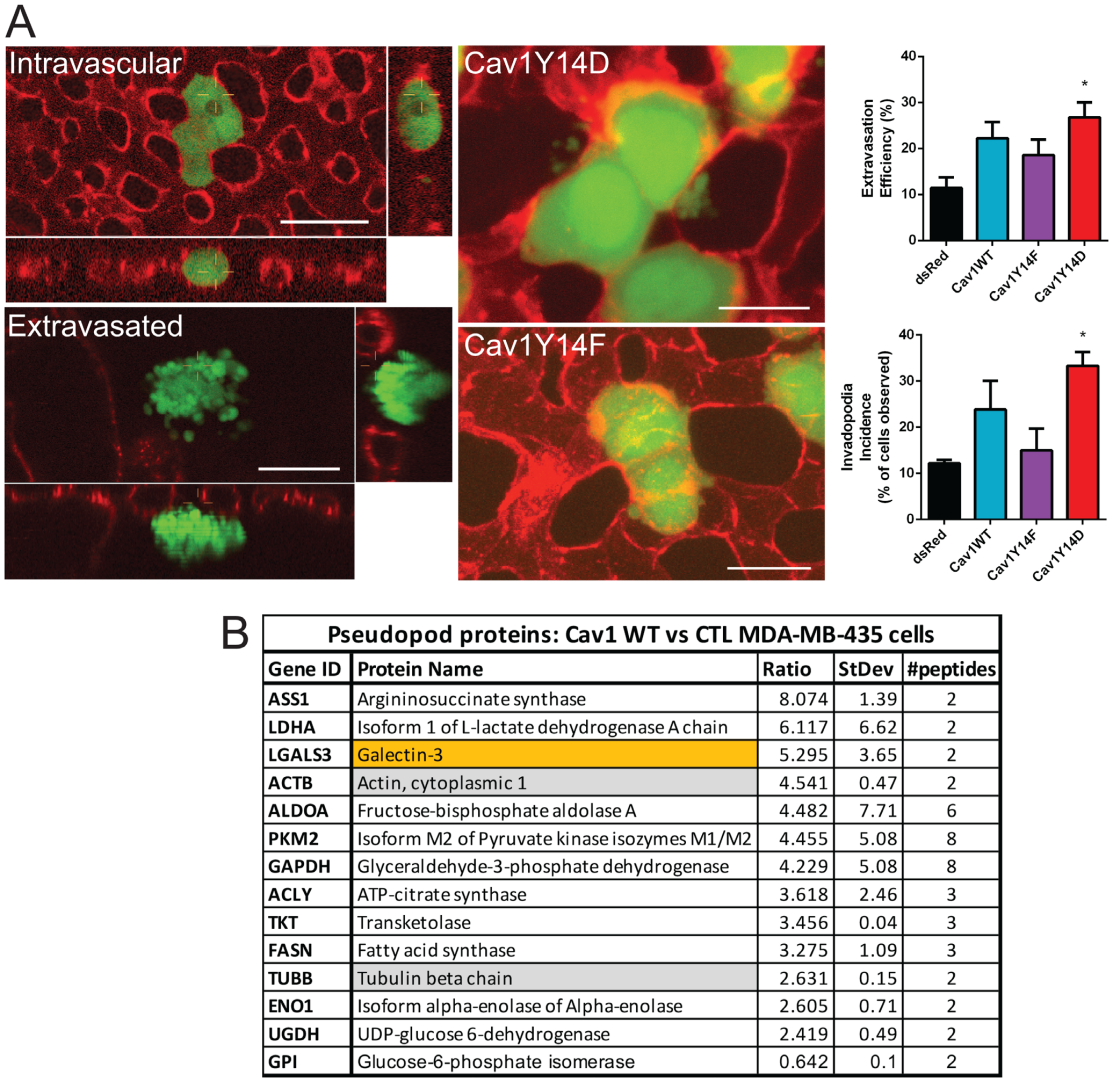
pCav1 promotes invadopodia formation and tumor cell invasion *in vivo*. (**A**) GFP lentiviral infected MDA-MB-435 stable cells were intravenously injected into the chorioallantoic membrane of avian embryos (representative images of Cav1Y14F and Cav1Y14D cells are shown). Intravital images were acquired at T = 4 hrs post-IV injection of cells. Lectin-rhodamine (red) labels the luminal surface of all endothelial cells. Graphs show extravasation efficiency (% of injected cells that successfully extravasate) and invadopodia incidence (% of cells that present extravascular protrusions) of MDA-MB-435 cells expressing dsRed, Cav1WT, Cav1Y14F or Cav1Y14D (±SEM; *n* = 3; ^*^ *p* < 0.05, ANOVA; Scale bar: 10 μm). (**B**) Quantitative proteomic analysis of pseudopod and cell body fractions from Cav1WT expressing MDA-MB-435 vs MDA-MB-435 cells identified 11 glycolytic enzymes, actin, tubulin and galectin-3 to be enriched in the pseudopodia of the Cav1 expressing cells (See Supplementary Table 1).

Expression of Cav1Y14D in MDA-MB-435 cells is associated with increased *in vitro* tumor cell migration and invasion [15]. In light of the dramatic tumor growth inhibition of Cav1Y14D MDA-MB-435 cells, we undertook to test whether these cells retained invasive capability *in vivo* by using time-lapse intravital imaging after i.v. injection of GFP-labeled Cav1Y14 mutant MDA-MB-435 cells into the vitelline vein of *ex ovo* chicken embryos preinjected with lectin-rhodamine [28]. The proportion of cells that successfully underwent trans-endothelial migration was quantified in multiple regions of interest (ROIs) at t = 0 and at t = 24 hrs post-injection. Cav1 WT overexpressing MDA-MB-435 cells showed enhanced extravasation from the bloodstream, and an increased incidence of extravascular invadopodia relative to dsRed MDA-MB-435 cells, as did Cav1Y14D relative to Cav1Y14F MDA-MB-435 cells (Figure 4). Although Y14 phosphorylation of Cav1 was associated with inhibition of cell proliferation and tumor growth, the capacity for invasion, assessed here based on expression of Cav1 WT relative to non-phosphorylatable Cav1Y14F and of the Cav1Y14D phosphomimetic, was retained.

Subcellular compartmentalization of glycolysis enzymes plays an important role in cell motility, presumably via regulation of local ATP and actomyosin behavior [29]. Glycolysis in filopodia and lamellipodia appears to be the primary bioenergetic pathway for cell motility in cancer cells and glycolytic enzymes accumulate in tumor pseudopodia [30, 31]. To assess whether Cav1 impacts local glycolysis in pseudopodia, we performed quantitative proteomic analysis of isolated pseudopodia [32] from control and Cav1 WT transfected MDA-MB-435 cells. We saw a dramatic enrichment of multiple glycolytic enzymes in the purified pseudopod fractions of Cav1 expressing MDA-MB-435 cells (Figure 4B). This is consistent with the Cav1-dependent local enrichment of glycolytic activity in pseudopod domains.

Global metabolic profiles observed for Cav1Y14D cells are consistent with reduced proliferation and elevated TP53, but may not reflect local non-oxidative glycolysis associated with invadopodia formation and invasion. The metabolic phenotype observed for Cav1Y14D tumor cells is consistent with TP53-associated reversal of the Warburg type-effect, reducing glucose uptake into glycolysis, hexosamine biosynthesis pathway (HBP) and nucleotide biosynthesis, while promoting carbon flux to oxidative respiration, DNA damage repair and resistance to oxidative stress [33]. Glycolysis and the hexosamine biosynthesis pathway (HBP) compete for fructose-6-phosphate [34], and in Cav1Y14D tumor cells, glycolysis is favored while HBP intermediates and UDP-GlcNAc are reduced. The increased motility of Cav1Y14D MDA-MB-435 cells suggests that Cav1-dependent local enrichment of glycolytic enzymes in pseudopodia contribute to invadopod protrusion and tumor cell invasion independently of TP53-induced metabolic changes.

UDP-GlcNAc is a rate limiting substrate for protein N-glycosylation, modifications to growth factor receptors and nutrient transporters that promote cell surface stability [35]. It is possible that pCav1 dependent upregulation of TP53 suppresses HBP and alters sensitivity to extracellular growth factors and nutrient uptake. Cav1 tumor suppressor activity is conditional on expression of galectin-3 and receptor N-glycosylation while pCav1 acts in concert with galectin-3 to promote FA turnover and tumor cell migration and invasion [17, 18, 36]. The Cav1 MDA-MB-435 pseudopod proteome included galectin-3 (Figure 4B) supporting a role for local pCav1-galectin-3 signaling to drive invadopod protrusion and tumor cell invasion.

## DISCUSSION

Our data reveal that pCav1 plays dual and opposing roles in tumor progression. High levels of pCav1 as indicated by Cav1 Y14D MDA-MB-435 expression inhibited tumor growth. The longer tumor latency of Cav1 WT tumors relative to Cav1 Y14F tumors also support a role for pCav1 in early stages of growth. The dramatic effects observed here for Cav1Y14D on tumor growth presumably reflects a phenotype related to excessively high pCav1 levels, that are not observed here in Cav1WT cells, but could conceivably occur down-stream of oxidative or mechanical stress [13, 22, 37]. While Cav1Y14D increases TP53 levels and suppresses tumor growth, extravasation and invadopodia protrusion remain active *in vivo.* Actomyosin dynamics in motile cells requires ATP production from glycolysis for microfilament remodeling. With conflicting requirements for resources, tumor cell proliferation and motility may be regulated to optimize different metabolic pathways depending on extracellular conditions and nutrient availability [38]. Our data suggest that Y14 phosphorylated Cav1 is a driver of metabolic control of both tumor cell proliferation and tumor cell motility and invasion.

## MATERIALS AND METHODS

### Cell culture, cell growth analysis and western blot and immunofluorescence

MDA-MB-435 cells and stable pools of cells transfected with Cav1WT, Cav1Y14F or Cav1Y14D [15, 22] were maintained in RPMI-1640 supplemented with 10% FBS, 100 IU/mL penicillin, 100 μg/mL streptomycin, 2 mmol/L L-glutamine and 25 mmol/L HEPES buffer at 37°C in 5% CO_2_/95% air incubator. Cell lines used were mycoplasma free and their authenticity confirmed by single tandem repeat analysis at the Centre for Applied Genomics (SickKids, Toronto, Canada). Cells seeded on 96-well plates were incubated in an IncuCyte ZOOM (Essen Biosciences) for 5 days and images captured every hour. Confluence in each well was determined using the Basic Analyzer of the IncuCyte ZOOM software, and doubling times were determined using exponential regression in Microsoft Excel. Protein extraction, western blots, immunofluorescence and pseudopod purification were as described [32]. The anti-pCav1 antibody crossreacts with p-paxillin [39] and we show only the 21 kD band corresponding to pCav1. Fluorescent images were quantified using ImagePro and densitometric analysis of western blots using ImageJ.

### Pseudopod proteomics

Pseudopodia were purified from MDA-MB-435 and Cav1 transfected MDA-MB-435 cells as previously described [40]. Samples were prepared for mass spectrometry as described [41]. For proteome profiling by relative quantification, binary analysis between time-points was performed by reductive dimethylation of primary amines in peptides using either light (CH_2_O) or heavy (CD_2_O) isotopologues of formaldehyde. Samples were analyzed on a linear trapping quadrupole-Orbitrap hybrid mass spectrometry (ThermoFisher Scientific, Waltham, MA, USA). Proteins were identified and quantified using Proteome Discoverer (v1.2, ThermoFisher).

### Xenograft tumor studies

Human MDA-MB-435 Cav1 Y14 mutant stable carcinoma cell lines were mixed with an equal volume of Matrigel (BD Biosciences, NJ, USA) prior to injection of 5x10^5^ cells subcutaneously into the right flank of 5 female nude mice (4-week-old NCr nude/Tac #NCRNU) under isofluorane anesthesia. All animal care protocols were followed in the care and treatment of the mice. Tumor measurements, weight and health condition were recorded. H&E staining and TP53 labeling were performed on paraffin embedded sections (Wax-It Histology Services, Vancouver, BC, Canada). DAB intensity for TP53 was quantified using Image J.

### Metabolite analysis

Cells were seeded in 6-well plate in replicates. After 24 h, the media were removed and the cells were washed 2-times with warm PBS, then the cells were placed on dry ice. The metabolites were immediately extracted by adding 1 mL of extraction solution (40% acetonitrile, 40% methanol, and 20% water) containing internal standards (500 µg/ml and 300 µg/ml of D7-Glucose and ^13^C^15^N-Tyrosine, respectively) and then the cells were scraped and collected in 1.5 mL vials. The mixture was shaken for 1 hr at 4°C and 1400 rpm in a Thermomixer (Eppendorf, Germany). The samples were spun down at 14000 rpm, for 10 min at 4°C (Eppendorf, Germany), and supernatants were transferred into fresh tubes to be evaporated to dryness in a CentreVap concentrator at 40°C (Labconco, MO, USA). The dry extract samples were stored at -80°C until LC/MS analysis. The dry metabolite extracts were reconstituted in 100 µL of water. The sample was injected twice through the HPLC (Dionex Corporation, CA, USA) in gradient reversed phase column (Inertsil ODS-3, 4.6 mm internal diameter, 150 mm length, and 3 µM particle size for positive and negative mode analysis). In positive mode analysis, the mobile phase gradient ramped from 5% to 90% of acetonitrile in 16 min, remained for 1 min at 90%, then returned to 5% acetonitrile in 0.1% acetic acid over two min. In negative mode, the acetonitrile composition ramped from 5 to 90% in 10 min, remained for 1 min at 90%, then returned to 5% acetonitrile in buffer-A (0.1% tributylamine, 0.03% acetic acid, 10% methanol). The total runtime in both modes was 20 min, the samples were stored at 4°C, and the injection volume was 10 µL. An automated washing procedure was developed before and after each sample to avoid any sample carryover.

The eluted metabolites were analyzed at the optimum polarity in MRM mode on electrospray ionization (ESI) triple-quadrupole mass spectrometer (ABSciex 5500 Qtrap, Toronto, ON, Canada). The mass spectrometric data acquisition time for each run was 20 min, and the dwell time for each MRM channel was 10 ms. Common mass spectrometric parameters are the same as tuning conditions previously described [42], except: GS1 and GS2 were 50 psi; CUR was 20 psi, and CAD was 3 and 7 for positive and negative modes, respectively, and source temperature (TEM) was 400°C. Signal was normalized to internal standard and cell number.

### *In vivo* extravasation experiments

Extravasation of human MDA-MB-435 Cav1 Y14 mutant stable carcinoma cell lines injected into the chorioallantoic membrane (CAM) of Day 13 chicken embyros was as described in [28].

### Statistical analyses

Data are presented as standard error mean (SEM). *p* values were obtained using one-way ANOVA.

## SUPPLEMENTARY MATERIALS


