# Supplementary material for: Caveolin-1 Y14 phosphorylation suppresses tumor growth while promoting invasion
Source: Oncotarget. 2019 Nov 19;10(62):6668–77. doi: 10.18632/oncotarget.27313 (PMC6877104; doi:10.18632/oncotarget.27313)
Supplement: Supplementary file 1 [file oncotarget-10-6668-s001.pdf]

Caveolin-1 Y14 phosphorylation suppresses tumor growth while promoting invasion

SUPPLEMENTARY MATERIALS

Supplementary Table 1: The Cav1-dependent MDA-MB-435 pseudopod proteome

Proteins with high ratios are enriched in pseudopodia of Cav1 transfected 435 relative to pseudopodia of WT 435 cells

	435 overexpressing Cav PS vs 435 WT Cav PS		
**Description**	**Ratio**	**StDev**	**# peptides**
emPAI:0.17 Tax_Id=9606 Gene_Symbol=TCP1 T-complex protein 1 subunit alpha	13.256	10.83	3
emPAI:0.57 Tax_Id=9606 Gene_Symbol=SFN Isoform 1 of 14-3-3 protein sigma	12.776	8.56	2
emPAI:0.46 Tax_Id=9606 Gene_Symbol=HSP90AA1 Isoform 1 of Heat shock protein HSP 90-alpha	12.066	5.17	2
emPAI:0.36 Tax_Id=9606 Gene_Symbol=PARK7 Protein DJ-1	9.797	7.79	9
emPAI:0.88 Tax_Id=9606 Gene_Symbol=EEF1A1 Elongation factor 1-alpha	8.277	8.9	2
emPAI:0.46 Tax_Id=9606 Gene_Symbol=SOD1 Superoxide dismutase [Cu-Zn]	8.219	6.22	6
emPAI:0.13 Tax_Id=9606 Gene_Symbol=ASS1 Argininosuccinate synthase	8.074	1.39	2
emPAI:0.41 Tax_Id=9606 Gene_Symbol=HSP90AB1 Heat shock protein HSP 90-beta	7.81	1.21	2
emPAI:0.41 Tax_Id=9606 Gene_Symbol=LDHA Isoform 1 of L-lactate dehydrogenase A chain	6.117	6.62	2
emPAI:0.13 Tax_Id=9606 Gene_Symbol=LGALS3 Galectin-3	5.295	3.65	2
emPAI:0.98 Tax_Id=9606 Gene_Symbol=ACTB Actin, cytoplasmic 1	4.541	0.47	2
emPAI:0.17 Tax_Id=9606 Gene_Symbol=ALDOA Fructose-bisphosphate aldolase A	4.482	7.71	6
emPAI:0.55 Tax_Id=9606 Gene_Symbol=PKM2 Isoform M2 of Pyruvate kinase isozymes M1/M2	4.455	5.08	8
emPAI:0.20 Tax_Id=9606 Gene_Symbol=HSPA8 Isoform 1 of Heat shock cognate 71 kDa protein	4.361	3.73	6
emPAI:1.20 Tax_Id=9606 Gene_Symbol=GAPDH Glyceraldehyde-3-phosphate dehydrogenase	4.229	5.08	8
emPAI:0.31 Tax_Id=9606 Gene_Symbol=PSAT1 Isoform 2 of Phosphoserine aminotransferase	3.675	1.93	2
emPAI:0.21 Tax_Id=9606 Gene_Symbol=PPA1 Inorganic pyrophosphatase	3.652	1.59	4
emPAI:0.03 Tax_Id=9606 Gene_Symbol=IARS IARS protein	3.635	0.49	2
emPAI:0.05 Tax_Id=9606 Gene_Symbol=ACLY ATP-citrate synthase	3.618	2.46	3
emPAI:0.17 Tax_Id=9606 Gene_Symbol=CCT5 T-complex protein 1 subunit epsilon	3.496	2.49	5
emPAI:0.28 Tax_Id=9606 Gene_Symbol=AHSA1 Activator of 90 kDa heat shock protein ATPase homolog 1	3.469	3.25	3
emPAI:0.15 Tax_Id=9606 Gene_Symbol=TKT Transketolase	3.456	0.04	3
emPAI:0.57 Tax_Id=9606 Gene_Symbol=ARF1 ADP-ribosylation factor	3.363	0.16	2
emPAI:0.11 Tax_Id=9606 Gene_Symbol=FASN Fatty acid synthase	3.275	1.09	3
emPAI:0.07 Tax_Id=9606 Gene_Symbol=FDPS Putative uncharacterized protein	3.225	1.26	3
emPAI:0.97 Tax_Id=9606 Gene_Symbol=UBC;UBB;RPS27A ubiquitin and ribosomal protein S27a precursor	3.073	1	3
emPAI:0.07 Tax_Id=9606 Gene_Symbol=USP5 Isoform Long of Ubiquitin carboxyl-terminal hydrolase 5	2.716	0.64	2
emPAI:0.67 Tax_Id=9606 Gene_Symbol=TUBB Tubulin beta chain	2.631	0.15	2
emPAI:0.83 Tax_Id=9606 Gene_Symbol=ENO1 Isoform alpha-enolase of Alpha-enolase	2.605	0.71	2
emPAI:0.36 Tax_Id=9606 Gene_Symbol=EIF5A Isoform 2 of Eukaryotic translation initiation factor 5A-1	2.57	1.9	2
emPAI:0.28 Tax_Id=9606 Gene_Symbol=HSP90B1 Endoplasmin	2.568	0.27	2
emPAI:0.06 Tax_Id=9606 Gene_Symbol=GANAB Isoform 2 of Neutral alpha-glucosidase AB	2.535	1.87	7
emPAI:0.19 Tax_Id=9606 Gene_Symbol=UGDH UDP-glucose 6-dehydrogenase	2.419	0.49	2
emPAI:1.86 Tax_Id=9606 Gene_Symbol=GSTM3 Glutathione S-transferase Mu 3	2.345	0.09	7

emPAI:0.23 Tax_Id=9606 Gene_Symbol=EIF4A1 Eukaryotic initiation factor 4A-I	1.483	0.09	2
emPAI:0.32 Tax_Id=9606 Gene_Symbol=SGTA Small glutamine-rich tetratricopeptide repeat-containing protein alpha	2.058	1.18	2
emPAI:0.12 Tax_Id=9606 Gene_Symbol=LAP3 Isoform 1 of Cytosol aminopeptidase	2.055	0.19	2
emPAI:0.07 Tax_Id=9606 Gene_Symbol=VCP Transitional endoplasmic reticulum ATPase	2.028	0.61	3
emPAI:0.14 Tax_Id=9606 Gene_Symbol=PSMD11 Proteasome 26S non-ATPase subunit 11 variant (Fragment)	1.865	0.3	3
emPAI:3.93 Tax_Id=9606 Gene_Symbol=GSTP1 Glutathione S-transferase P	1.567	0.42	10
emPAI:0.10 Tax_Id=9606 Gene_Symbol=EZR Ezrin	1.328	0.57	2
emPAI:0.09 Tax_Id=9606 Gene_Symbol=STRAP Serine-threonine kinase receptor-associated protein	1.285	0.59	8
emPAI:0.33 Tax_Id=9606 Gene_Symbol=TPT1 Tumor protein, translationally-controlled 1	1.196	0.25	3
emPAI:0.20 Tax_Id=9606 Gene_Symbol=ARPC2 Actin-related protein 2/3 complex subunit 2	1.189	0.23	2
emPAI:0.40 Tax_Id=9606 Gene_Symbol=ACTR3 Actin-related protein 3	1.098	0.78	4
emPAI:0.90 Tax_Id=9606 Gene_Symbol=RPLP0 60S acidic ribosomal protein P0	0.993	0.06	3
emPAI:0.25 Tax_Id=9606 Gene_Symbol=P4HB Protein disulfide-isomerase precursor	0.979	0.24	3
emPAI:0.19 Tax_Id=9606 Gene_Symbol=ANXA5 Annexin A5	0.816	0.12	2
emPAI:0.22 Tax_Id=9606 Gene_Symbol=EEF1A1 Elongation factor 1-alpha	0.778	0.22	2
emPAI:0.19 Tax_Id=9606 Gene_Symbol=HSPA5 HSPA5 protein	0.774	0.17	3
emPAI:0.06 Tax_Id=9606 Gene_Symbol=MAP4 Isoform 2 of Microtubule-associated protein	0.729	0.14	2
emPAI:0.52 Tax_Id=9606 Gene_Symbol=PRDX5 Isoform Mitochondrial of Peroxiredoxin-5, mitochondrial precursor	0.724	0.12	2
emPAI:0.05 Tax_Id=9606 Gene_Symbol=TLN1 Talin-1	0.684	0.31	3
emPAI:0.13 Tax_Id=9606 Gene_Symbol=MTHFD1 C-1-tetrahydrofolate synthase, cytoplasmic	0.659	0.15	2
emPAI:0.46 Tax_Id=9606 Gene_Symbol=PRDX6 Peroxiredoxin-6	0.661	0.34	2
emPAI:0.56 Tax_Id=9606 Gene_Symbol=CCT2 T-complex protein 1 subunit beta	0.654	0.24	7
emPAI:0.09 Tax_Id=9606 Gene_Symbol=MYH9 Myosin-9	0.65	0.28	5
emPAI:0.31 Tax_Id=9606 Gene_Symbol=EEF2 Elongation factor 2	0.639	0.31	5
emPAI:0.22 Tax_Id=9606 Gene_Symbol=GPI Glucose-6-phosphate isomerase	0.642	0.1	2
emPAI:1.77 Tax_Id=9606 Gene_Symbol=PPIA;PPIAL3;LOC654188 Peptidyl-prolyl cis-trans isomerase A	0.607	0.24	3
emPAI:0.38 Tax_Id=9606 Gene_Symbol=YWHAE 14-3-3 protein epsilon	0.578	0.01	2
emPAI:1.24 Tax_Id=9606 Gene_Symbol=PFN1 Profilin-1	0.581	0.13	3
emPAI:0.64 Tax_Id=9606 Gene_Symbol=CCT6A T-complex protein 1 subunit zeta	0.573	0.08	7
emPAI:0.08 Tax_Id=9606 Gene_Symbol=SERPINB1 Leukocyte elastase inhibitor	0.542	0.2	2
emPAI:0.56 Tax_Id=9606 Gene_Symbol=TUBB2C Tubulin beta-2C chain	0.508	0.3	2
emPAI:0.46 Tax_Id=9606 Gene_Symbol=TUBA1C Tubulin alpha-1C chain	0.475	0.12	5
emPAI:0.21 Tax_Id=9606 Gene_Symbol=PAICS Multifunctional protein ADE2	0.451	0.09	2
emPAI:0.29 Tax_Id=9606 Gene_Symbol=EEF1G Elongation factor 1-gamma	0.427	0.04	2
emPAI:0.52 Tax_Id=9606 Gene_Symbol=PRDX1 Peroxiredoxin-1	0.395	0.01	2
emPAI:0.11 Tax_Id=9606 Gene_Symbol=DPYSL2 Dihydropyrimidinase-related protein 2	0.384	0.05	2
emPAI:0.20 Tax_Id=9606 Gene_Symbol=CORO1A Coronin-1A	0.381	0.18	3
emPAI:0.05 Tax_Id=9606 Gene_Symbol=CLTC Isoform 1 of Clathrin heavy chain 1	0.345	1.04	3

Complete list of proteins identified by quantitative mass spectroscopy showing enrichment in purified pseudopodia of Cav1 WT-expressing MDA-MB-435 cells relative to pseudopodia of control MDA-MB-435 cells. Data represent 3 independent experiments. 3/1: ratio of detected protein in pseudopodia of Cav1 WT vs control MDA-MB-435 cells; StDev: standard deviation over 3 experiments; # peptides: number of detected peptides that match the protein.
